# Centring participant experience: a realist evaluation of a menstruator-friendly facility design project in a refugee settlement, Lebanon

**DOI:** 10.1186/s12905-024-02961-z

**Published:** 2024-03-09

**Authors:** Georgia Hales, Paul Hutchings, Katy Roelich, Mahua Das, Alexandra Machado, Debora Bonucci, Farah Salem

**Affiliations:** 1 University of Leeds, Leeds, UK; 2 International Federation of Red Cross and Red Crescent Societies (IFRC) CH, Geneva, Switzerland; 3 British Red Cross, London, UK

**Keywords:** Menstrual health and hygiene, Lebanon, Refugee settlement, Realist evaluation, Participation, MHM-facility design

## Abstract

**Introduction:**

Menstrual health in humanitarian contexts is a neglected topic. Its taboo nature presents difficulties for participants in menstrual health projects in these particularly challenging settings. Namely, their experiences may be concealed or overlooked in projects that are typically outcome focused. Realist Evaluation is a useful method to unearth and explore the hidden mechanisms and their causes, which lead to positive or negative participant experiences. The authors have applied this approach to a robust humanitarian menstrual health project to explore how to centre the emotional wellbeing of participants at all stages: prior to, during, and post-participation.

**Study setting:**

The project studied was led by the International Federation of Red Cross and Red Crescent Societies who piloted their adaptable manual for menstruator–friendly water, sanitation and hygiene (WaSH) facility design in humanitarian contexts. It was conducted by the Lebanese Red Cross in an informal tented settlement hosting Syrian refugees in Qaa, Lebanon.

**Methods:**

The authors collected interview and focus group data on the contextual factors and processes within the project from nine project staff and 16 settlement inhabitants. They used a realist process of theory development, testing, and consolidation to understand how and under what circumstances the project inputs affected participants’ wellbeing.

**Results:**

The contextual factors and causal mechanisms promoting participant experience comprised individual (choices influencing and experience during participation), interpersonal (group dynamics and the role of non-menstruators), and organisational (expertise and knowledge, relationship to participants and cultural differences) factors.

**Implications:**

The research uses a case study from a renowned humanitarian organisation who provided a well-delivered project in a conducive environment to explore the mechanisms and contexts that can promote wider learning and refine understanding and programming in this under-researched and -theorised space. Specifically, it informs which contextual factors and project inputs must be present within a menstrual health project to ensure participant satisfaction whilst efficiently delivering well-designed menstruator-friendly WaSH facilities.

## Terms

To be inclusive of gender diverse persons Hennegan et al.’s [9] definition of menstrual health for policy, practice, and research defines those who have the ability to menstruate as ‘menstruators’ and those who do not as ‘non-menstruators’. A significant shift in collective language will pose challenges. In the United Kingdom (UK) Dahlen [4] writes how gender-neutral terminology in medical literature faced backlash in fear of the erasure of women’s needs. Different cultures have varying views on gender identity meaning some languages may not offer gender-neutral terms or be able to translate new terms from one language to another. Thus, identifying menstruators and non-menstruators across different humanitarian settings may result in people being left out. To avoid this we can use gender-additive language where both gendered and gender-neutral language is used e.g. ‘women, girls, and menstruators’ as is demonstrated in a UK National Health Service Trust guide to ‘Gender Inclusive Language In Perinatal Services’ [7]. In this paper, we use the term menstruator, however quotes from interviews and the literature still use gendered binary terms 'women' and 'girls'.

## Background

Poor access to menstrual health—comprising education, materials, water, sanitation and hygiene (WaSH) facilities, disposal methods, healthcare, a supportive environment, and the choice to participate in daily activities—is a global issue [9]. Menstruation is documented internationally as a stigmatised and taboo topic meaning conversations around the subject are often either wrongly informed, minimal or non-existent [6]. In the absence of a clear and open debate, WaSH services may not consider menstrual health, and fail to deliver menstruator-friendly, culturally appropriate WaSH facilities [24]. A lack of or inappropriate solutions may cause shame, stress, exhaustion, fear, embarrassment, stigma, loss of dignity, and Gender-Based Violence (GBV); since Menstrual Hygiene Management (MHM) requires privacy, menstruators often choose to use WaSH facilities at night, leaving them susceptible to attack and sexual assault [10]. These issues are exacerbated in humanitarian settings due to overcrowding, decreased lack of facilities and materials, and safety issues [18]. The UNHCR [28] estimates that 110 million people (1.4% of the global population) are currently forcibly displaced – of these 29 million are menstruators. Therefore, MHM in humanitarian settings is a significant challenge that needs to be addressed urgently.

Current menstrual health guidance from NGOs advocates for the consultation of menstruators on their needs before implementing a menstrual health project [18, 23]. Additionally, in humanitarianism, general opinion is that humanitarian action is ‘best developed with and for affected people’ [33], p. 13). By shaping projects around local sociocultural, economic and political situations, listening to people’s needs and utilising local knowledge, skills and resources, results are set up to be more appropriate and thus more sustainable [21]. However, there is limited empirical evidence on the efficacy of participatory approaches in menstrual health projects, the impact on the lives of participants, and uncertainty about the extent to which they are implemented in practice [30].

Even when participatory approaches are implemented, a 2020 UNHCR study found many barriers to internally displaced menstruators’ participation including preoccupation with meeting safety and survival needs, GBV, consultation fatigue, and a negative reaction from non-menstruators [1]. When menstruators are able and willing to participate, gender-related development projects have historically overburdened them through adding to their triple role (childcare, labour, community work), disturbing power relations, or being extractive, [20]. Accordingly, the UNHCR (2020) study also found that ‘participation is not always empowering for [displaced] women and girls’ and that participatory interventions ‘can unintentionally disempower [them] and reinforce the dominance of men’. In the context of menstrual health projects, menstruators may be dissuaded from taking part due to the stigmatised and taboo nature of the topic. If they are involved, they may be asked personal, extractive, and potentially triggering questions. Following participation, they may face backlash from family or community members [1].

It is a well-documented need for participation to be respectful, non-coercive, non-intrusive, alongside aiming to mitigate unplanned negative outcomes, wherein the project respects the rights, needs, and perspectives of the individuals it aims to serve [31]. The global literature on participatory research approaches emphasises the need to involve individuals affected by the project in the design, implementation, and evaluation processes to ensure the intervention is ethical, culturally sensitive, and addresses the needs of the population [13, 16, 19]. The standardisation of humanitarian action supports this. In terms of accountability to affected populations, the Sphere Standards underscore the importance of communication, participation, and feedback mechanisms (Sphere [25]. The Humanitarian Accountability Partnership’s Framework provides indicators for ensuring accountability, stressing the need for the participation of affected populations in decision-making processes [8]. The authors used these principles to theorise how to achieve a universally positive participatory experience wherein the benefits of participation are balanced with the burden. In this way, the authors reason that a project's success is not solely measured by its outcomes but also by the thoughtful process through which those outcomes are achieved. Thus, the aim of this study was to look at how we can centre the experiences of menstruators at all stages—pre- during and post-participation—of menstrual health projects in humanitarian settings.

There are varying degrees of participation, illustrated by Arnstein’s [2] ladder of citizen participation wherein each rung represents increasing levels of agency and power, from one – manipulation—to eight – citizen control. In the project studied in this paper, the level of participation was midway at number four – consultation – where participants’ opinion was invited through multiple rounds of Focus Group Discussions (FGDs) with iterative feedback on facility designs. The International Federation of Red Cross and Red Crescent Societies’ (IFRC) project was to pilot a menstruator-friendly WaSH facility design manual [12] that can be adapted to geographical and cultural requirements and rapidly deployed in humanitarian contexts. This is to be used alongside their guideline for consulting the community on their menstrual needs [11], with the manual providing appropriate options based on their responses. Please see the references for online links to both of these.

## Methods

### Research aim, framing and approach

The aim of the research was to answer the question: How can we centre participant experience at all stages of menstrual heath projects in humanitarian settings? The authors do this by using a Realist Evaluation to unearth the hidden causal forces (or mechanisms) that promote positive—or avoid negative—experiences for participants, which may be concealed or overlooked when implementing projects that are purely outcome-focused. Realist Evaluation is an established analytical approach based on the notion that interventions have different outcomes depending on how their inputs interact with the context in which it takes place [22, 32]. It asks how and why interventions work, for whom, and in what circumstances by understanding the Context-Mechanism-Outcome (CMO) configurations at play. This allows insights into the contextual factors that are conducive to menstrual health projects when implementing or scaling up elsewhere. The authors developed interview questions for nine project staff and FGDs with 16 Syrian inhabitants of an informal tented settlement in Lebanon to explore theories around how projects such as these can promote a positive participant experience. The Socio-Ecological Model—a framework that can be used to understand how interventions work within different interacting systems in relation to the individual—provided a useful framing for designing questions targeted at the different levels to understand their influence on the individual, the project, and each other [15].

### Study population and setting

The study was on a long-term informal tented settlement in Qaa, Northeastern Lebanon, hosting 50 Syrian refugees. Qaa is a hard to reach border area with over 100 informal tented settlements, where access for international humanitarian actors has long been a challenge. The Lebanese Red Cross (LRC) has managed to develop a cohesive and positive relationship with the Syrian communities. This specific site was chosen due to the number of menstruators who would be able to take part, good road access, having built rapport with the community previously, and them being regarded as receptive to a menstrual health project. Data included Key Informant Interviews (KIIs) with at least one staff member from each of the four organisations involved in the project: LRC, British Red Cross (BRC), IFRC, and ARUP. The LRC recruited settlement inhabitants to partake in five FGDs through community meetings. All menstruators (total 13) were included within four FGDs; no-one refused to take part. Their age range was between 18 and 42. Eleven were married, one widowed and one unmarried. Only three adult non-menstruators took part in the final FGD as the rest had work commitments. They were all in their 30s and married. All participants were cisgender. This information is summarised in Table 1.

**Table 1:** Number of interviewees and focus group discussion participants

Participant	Session type	Group no	# Participants	TOTAL
LRC Social Systems WaSH officer	KII	N/A	1	9
LRC Technical Systems WaSH officer	KII	N/A	1	
LRC field staff	KII	N/A	3	
ARUP WaSH consultant	KII	N/A	1	
ARUP structural engineer	KII	N/A	1	
BRC project manager	KII	N/A	1	
IFRC project manager	KII	N/A	1	
Menstruator settlement inhabitant	FGD	1	4	13
Menstruator settlement inhabitant	FGD	2	3	
Menstruator settlement inhabitant	FGD	3	3	
Menstruator settlement inhabitant	FGD	4	3	
Non-menstruator settlement inhabitant	FGD	N/A	3	3

### Procedure

Though the intervention studied in this paper was a project, Realist Evaluations call for the development of programme theories, thus the authors refer to them as such. The first step was to develop Initial Programme Theories (IPTs) at different levels of the Socio-Ecological Model: individual, community, and organisational. These were drawn from understandings from the literature, project document review, and preliminary conversations with Red Cross project managers. These IPTs aimed to explain why a project produces certain outcomes based on the interactions between the resources put into the project and the specific context. To test the IPTs, the authors developed a qualitative semi-structured interview protocol for KIIs with project staff and a proforma for settlement inhabitants. This approach to testing IPTs was similar to but not the same as the teacher-learner cycle wherein IPTs are placed before respondents for them to confirm, deny, or refine the theory [17]. By asking tailored questions, respondents were able to give more detailed and nuanced responses.

Due to the unstable political situation and risks associated with travel, the lead author undertook KIIs online via Microsoft Teams, using its live transcription tool. She had experience conducting KIIs with schoolteachers in India on menstrual health as part of her Master’s degree, and with water service providers in Cambodia where she worked as a WaSH consultant for 1.5 years. The KII and FGD questions were reviewed by the lead author’s three senior academic supervisors, and the LRC’s Social Systems WaSH Officer, who was able to confirm the guide’s contextual relevance and appropriateness. All four mentioned are co-authors to this paper. The FGDs with settlement inhabitants were conducted under the lead author’s instruction by the WaSH officer with responses recorded on a proforma. The WaSH officer had taken part in all available training courses from the LRC. Rapport was created between the lead author and the WaSH officer with coordination calls before and after the FGDs. The one-page instruction document outlined the target participants, how the groups were to be split, logistics of filling out the proforma, contact information, and ethical procedures. These included direction for participants to read (or have read to them) the Explanatory Statement provided and to sign the consent form, for the FGD facilitator(s) to sign a confidentiality form, and instruction on how to conduct sensitive FGDs as outlined in the University of Leeds ethical review. The questions for FGDs were written in English by the lead author. These were translated into and conducted in Arabic by the Social Systems WaSH Officer (fluent in English, native Arabic). She then back translated them into English. All KIIs apart from with field staff were conducted in English by the lead author. The group field staff KII was lead jointly by the lead author and the Social Systems WaSH Officer in a mixture of English and Arabic. The online KIIs and in-person FGDs took place between February and April 2022. The authors listened to the recordings and edited the transcripts from April to May 2022.

### Data analysis

Aligning to realist approaches, the authors were retroductive in their data analysis; applying inductive and deductive coding to both KII and FGD data whilst employing the lead author’s own reasoning to identify generative causation (The RAMESES II Project 2017). The two types of data were utilised in the same way to identify generative mechanisms. Following Dalkin et al.’s (2015) suggestion to aid clarification between context and mechanism, the mechanisms were broken down in their two constituent parts: ‘resource’, denoting the input of the project and ‘reasoning’, denoting how that resource interacts with the context. Thus, ‘MCMO’ (Mechanism(resource)-Context-Mechanism(reasoning)-Outcome) was used when refining the programme theory (PT). In many cases both KIIs and FGDs were used to code the same MCMO. The authors followed Gilmore et al.’s [5] approach for their data analysis method. It consisted of the following four steps:

#### Data preparation

The lead author transcribed KIIs and read the FGD proforma to gain a better contextual understanding. The authors uploaded each KII and FGD as one individual data source on excel. They then created a corresponding Mastersheet for all IPTs in which to align MCMOs as ‘evidence’ from the KIIs and FGDs.

#### MCMO extraction and elicitation

The lead author went through each data source individually and coded when an observable MCMO was found, which they either added to an existing IPT, or used to create a new PT by placing the quote next to the IPT/PT and associated MCMOs. Once a whole data source was read and coded the authors reviewed data again by refining the MCMOs, their IPT or PT and labelling whether this supported, refuted or lacked evidence to make conclusions on the IPT/PT.

#### Using MCMOs to refine PTs

The authors refined the IPTs/PTs continuously throughout data analysis and merged similar IPTs/PTs for simplicity.

#### Collating evidence and refinement verification

The authors found 20 MCMOs across the data sources. Once coding was complete, the authors collated all MCMOs and their supporting evidence into tables. These were compared with mechanisms found in the literature where relevant (as seen in the results section) and used to consolidate and define nine PTs.

## Results

Through testing and verifying the nine PTs through data collection and analysis the authors consolidated them into one overarching PT:

In a participatory project for menstruator-friendly WaSH facilities in humanitarian settings, the Red Cross demonstrated examples of how to centre participants’ experience at different stages when implementing their adaptable manual. Pre-participation they provide MHM education, clearly outline the project, and adapt FGDs around gendered responsibilities. To aid the experience during participation they create a safe space for FGDs, navigate group dynamics, and deliver MHM and FGD training and guidelines to field staff along with building rapport and bridging cultural gaps. To avoid negative consequences post-participation they negotiate how to incorporate participants’ needs into designs, and navigate social dynamics and non-menstruator support. These inputs and resources generate feelings of support, comfort, safety, trust, and confidence leading to participant satisfaction at most stages of the project alongside the outcome of appropriately designed menstruator-friendly WaSH facilities. The project also gave examples of when participant experience was not centred such as the failure to communicate project limitations resulting in disappointment and a break in trust between participant and staff.

The findings of the retroductive analysis of KII and FGD transcripts demonstrated that a complex interaction of individual, interpersonal, community, and organisational factors influenced participant experience and choice to participate before, during and after project participation. Individual-level factors were menstruators’ valuing of menstrual health, aided by MHM education and explanations of the project details prior to recruitment. Interpersonal factors consisted of non-menstruator and religious leader support for the project. Community factors were the splitting of demographic groups for FGDs. Organisational factors were cultivating good relationships with participants, minimising cultural gaps, adapting the project around gendered responsibilities, negotiating needs into designs, explaining limitations, and creating safe spaces for FGDs. When certain limitations were not met nor the reason for this communicated, participants were disappointed and disconcerted.

These drivers of participant experience led to the identification of nine mechanisms. These were: feeling informed, autonomous, and prepared (PT1); Valuing MHM (PT2); Feeling included and able to participate (PT3); Feeling familiar and trusting (PT4); Feeling safe (PT5); Feeling comfortable to share and heard equally (PT6); Adjusting expectations, accepting limitations and feeling respected and accounted for (PT7); Feeling valued, satisfied with outcomes, and confident in the project (PT8); Feeling supported and unthreatened by non-menstruators (PT9). The nine mechanisms are discussed presently, each denoted with a sub-theory of the consolidated programme theory above, explained by relationships to MCMOs. In realist evaluation, researchers often use a combination of informal conversations and observations, document review, literature review, and formal interviews or FGDs, which form and refine the programme theories through retroductive reasoning. For this reason, the PTs presented below are exemplified by supporting quotes from the KIIs and FGDs (italics, no reference) but are sometimes substantiated or substituted with evidence from the literature (upright, with reference).

### Pre-participation

#### PT1 – Informed consent

If the project is well explained to participants beforehand then they can make informed decisions to participate. They are also then prepared to discuss the sensitive topic of menstruation, avoiding surprise or discomfort, which allows them to feel contented enough to continue with the project. Participants agreed that project staff did well to *‘explain to us the details of the project’,* with one staff member reasoning, ‘*all this information were part of the mobilization [of] the community’.* Participants reported having no personal issues with taking part, with staff clarifying that *‘we're generally received with a good attitude towards it, so no one really refused to take part in this project. On the contrary, they were welcomed’.*

#### PT2 – MHM Education

If staff assess individual’s level of MHM knowledge and tailor a session on the importance of proper MHM, stigma will be reduced, which may have deterred community members from taking part. Additionally, individuals will understand the health implications of improper MHM. This leads all community members to value the project enough to participate meaning designs are representative of all community members. Moreover, everyone will have an adequate, equal level of MHM knowledge, allowing for greater awareness of options when considering facility design. Participants agreed that ‘*the project helped us learn about and improve our personal hygiene during menstruation’.* All menstruators chose to take part in the project, demonstrating their valuing or willingness to interact with the topic.

#### PT3 – Inclusion

If a project works around other duties and demographical barriers to participation such as gendered responsibilities, age, school, religion, disabilities, or different languages, then menstruators feel included and their voices represented. Menstruators’ ‘triple burden of roles’ may be exacerbated in times of displacement, especially within the early stages of an emergency, meaning there is less time and willingness to participate in projects [1, 20]. The menstruator roles in this community were typical (cooking, childcare, agricultural work). The LRC were conscious to work around this saying that, ‘*When we schedule our FGDs… we tend to take everyone’s different roles into consideration to try and make it a set date where everyone can participate and we tried to make them shorter because… people have to cook or to clean or to take care of the kids’.* When such a role would interfere with participation, the LRC would mitigate this, explaining that ‘*if someone has, for example, the baby that they can't leave, then the FGD we moved to their house if they allow us to’.* Participants reported that they were happy for this to happen and that their other responsibilities were not infringed upon nor were they prevented from participating.

### During participation

#### PT4 – Training, guidelines, rapport, and cultural gaps

If staff have rapport with the community and are trained and experienced in using IFRC’s guidelines to conduct sensitive MHM-related FGDs and bridge cultural differences then only relevant, tactful questions will be asked. Along with feeling comfortable, familiar and trusting, menstruators’ time and emotional triggers are minimised. One of the main aspects of the project attributing to the comfortability of participants was the relationship the field staff had with the community. The factors contributing to this were the continual rigorous training they receive on community engagement, the renown of the Red Cross, the years spent working together, and their minimal cultural differences between. Where cultural differences did exist, staff bridged these gaps sensitively. For example, the participants believed there to be no cultural differences regarding beliefs around menstruation, however this was due to staff not ‘*mak[ing] them feel like they are different or that they have a different knowledge’.* This allowed participants to openly discuss MHM without feeling judged or self-conscious. One staff member elucidates how the Red Cross differs from other organisations in this way:

*‘It's not the same [as] if [an]other international organisation… come to do… the participation because they don't understand the language, they don't understand… so the concept [of the] Red Cross is very different than other organisations… We are before the emergency happen[s], we are dealing [with] them urgently and we are after the emergencies in the community. We have many other organisations coming with the… backpacker. The level of our participation is always much higher because… the volunteers… are community volunteers and they have the same needs of the [community]. So, it's easier for us to understand the participation than [other] organisations... we are very [on] the ground… we have expertise in understanding the communities that perhaps other organisations lack.’*

#### PT5 – Safety

If the setting poses safety concerns to menstruators e.g. outsiders infiltrating the settlement or living in close proximity to one another, staff can create a safe menstruator-only space for FGDs where menstruators feel able and comfortable to discuss personal or sensitive experiences and opinions freely. The UNHCR found that displaced menstruators are ‘often preoccupied with meeting safety and survival needs that take time and energy away from participation’ (2020, p.8). Contextually, the settlement was already considered to be a ‘*kind of quiet’* and ‘*really safe place’* where the *‘landowner is friendly and camp is far away from road so deters thieves’*. Additionally, when asked if they feel safe to use facilities at night participants responded ‘*yes of course’*. Project staff worked to ensure that the FGDs were safe making them ‘*very small’*, and guaranteeing that ‘*there is no one that shouldn't be there that's looming around’.* Thus, safety did not pose an issue to their participation and everyone shared freely in the group.

#### PT6 – Group dynamics

If menstruation is not comfortably discussed between everyone, even if a community is familiar and of the same ethnicity and culture, FGDs can be split by age and sex, with same-sex facilitators, to promote comfort resulting in ease of open discussion where voices are heard equally. Group dynamics influence the ease at which people freely share within FGDs, and thus impact the efficacy of the exercise. This may be due to ‘the presence of a domineering and judgemental participant’, unfamiliarity or over-familiarity, and cultural differences (Scheelbeek et al., 2020). Thus, guidance suggests splitting groups by age, ethnicity and gender [18]. One staff member explains how it was relevant to *‘split the community between males and females to give everyone their complete privacy and… to assign a male volunteer to conduct the FGD with the males, because… it's… a little bit sensitive and a little bit embarrassing and… a topic that it's not generally discussed between men and women together’.* They were also split *‘for the comfort of the ease of communication basically between different age groups’.* One staff member expands: ‘*As comfortable as people may seem around each other, sometimes it might just be something between… a mother and a daughter… where they don't necessarily talk about everything with each other… then that allowed them to have… a space where they could talk among individuals closer to their age, going through the same things they're going through.’* In this instance the FGDs worked very well as* ‘most of the people know each other, most of them have been there together in the same space for a while so we didn't really notice any of these tensions. Most of these tensions are found in the larger [informal tented settlements] where… we have newcomers or people who came from different regions who don't necessarily know each other’.*

### Post-participation

#### PT7 – Negotiating limitations

If there are contextual limitations to what is possible in facility design, staff can explain and negotiate participants’ needs with them, thus avoiding disappointment and promoting feelings of acceptance and being respected and accounted for. They will understand that their suggestions have not been taken into account for good reason and will not feel unheard or that their participation was futile. Participants are then able to feel satisfied with the delivery of robust and culturally appropriate facilities. Without this, it may damage the trust that project staff worked hard to build. When some design constraints were communicated to participants, they were made to feel ‘*comfortable in general’* in that they ‘*had no problems because everything was explained to [them]’.* When limitations are not explained ‘harm… occurs when agencies engage with women and girls but do not meet their concerns, particularly if agencies do not set clear expectations when they convene consultations’ (Anderson, p.33, 2020). Udoewa [27] writes that Coloniality is inherent in participatory design, wherein the community members’ ‘disappointment is greater due to the greater expectations and presencing [a field of co-creation and social warmth] potential of a ‘participatory design’ process’ (p.1). An example here was that although participants attested that field staff explained the project to them well, one shortcoming was its failure to explain its limitations. Within the initial FGDs participants asked for greater access to sanitary pads, however this was outside the project’s scope. When it came to the research, the participants explained their disappointment in this need not being met. They elucidated,* ‘we informed the facilitators that we have a gap in our ability to purchase menstrual hygiene products… but until now, we still haven’t been able to fill that gap… sometimes we cannot practice [MHM] because we are not able to purchase menstrual pads’.*

#### PT8—Valuing participant input

If the engineers have expert knowledge and experience in the field and they value participants’ FGD responses then they will incorporate them well into the designs so that the facilities are adequately designed according to participants’ needs as well as technologically sound. Participants are then satisfied with the outcome and their time spent contributing to the project. The UNHCR finds that ‘consultation remains a largely passive mode of participation, especially when the persons consulted do not see or hear the outcomes of their time and input’ (Anderson, p.33, 2020). In this case, participants were able to clearly see how the engineers took their responses into consideration with the design of the facilities. While the literature and guidance suggest the importance of keeping participant’s time to a minimum to avoid consultation fatigue, the international engineers felt the more FGDs there were, the better [1]. Since they were not able to travel to the site, they felt that feedback from the FGDs allowed them to have a greater understanding of the context and could therefore inform designs that are more suitable.

#### PT9 – Non-menstruator support

If project staff work to understand the social dynamics of the community and ensure that non-menstruators are supportive of the project and of their menstruating family members taking part, then menstruators will feel no repercussive threat in participating and so choose to do so without any negative consequences. This PT relates to pre-, during, and post-participation as the attitudes and actions of non-menstruators might influence menstruator experience of participation at any stage of the project. We have chosen to put it under post-participation as we discuss the ongoing impact of non-menstruator influenceafter the project has finished. The LRC first engaged the Shaweesh (community leader) about the project who in turn encouraged menstruators to take part. Non-menstruators were also supportive of menstruator involvement in the project. Staff said they ‘*didn't sense any tension between the men and the women’* and that they *‘did not face any problems… on the contrary the men were welcoming the idea’.* They said the non-menstruators felt *‘the women can take part, should take part; it's interesting, it's good for them to gain the knowledge’.* Another goes on to explain that *‘it was a safe space because the community respects the fact that the project was being implemented with women and even the males that were the heads of households did not have any problem with the woman sitting alone with the facilitator’*. Considering post-project impacts, staff asked menstruators *‘do you think that the men will be able to leave this facility alone or would they want to try it as well… and everyone was in agreement saying that since this is a female facility they will respect this and they will leave it for the women’.*

In terms of the involvement of non-menstruators themselves, project staff explained that ‘*the attitude towards that is a little bit, it's not necessarily shameful, but kind of a taboo kind of something that is supposedly just for women “We don't want to be part of this conversation’’, *etc*.’*. They were, however, happy to partake in the project from a practical standpoint, with staff saying they were *‘very helpful when it comes to designs through different prototypes’.* They continue, ‘*when we first put the facility on field the men were there as well, and they were telling us, like, “yeah, this is good, it looks great” *etc*. but kind of giving us their feedback from the outside’.* This brings about the question for the need to engage non-menstruators in menstrual health projects when the attitudes and actions are ‘*not in a harmful way’* as demonstrated here, or—when they are a problem—how they can be best engaged. One staff member felt that *‘ideally, eventually, hopefully men and boys should be included in these types of projects, but [in] a very sensitive way of doing it because of the cultural considerations and of course, because some of them don't want to be involved’*.

## Discussion

The programme theory examined in this paper explores how the interacting interventions and contextual factors work to produce mechanisms, which lead to positive participant experience leading up to, during, and after participating in a menstrual health project. The authors used current literature on menstruator participant experience in menstrual health and humanitarian projects to guide data analysis and synthesis. This led to the development of a middle range theory in the form of nine programme sub-theories that explain how this can be achieved in this context. The distinguishing contribution of this research is its identification of the strategies and resources required to ensure menstruator participation is safe and satisfactory.

### Localisation of aid as an operational approach to centre participant experience

The results demonstrate how the project interacted with the organisational and local contexts. The IFRC is a globally renowned, decentralised humanitarian organisation who are able to be present at all stages of an emergency. The field staff of their national teams are generally of a similar culture and language as the communities they work with. These factors allow for great rapport and minimal cultural gaps, which contribute to feelings of ease and trust with the community. The humanitarian sector receives many international workers. This means there is often a dissonance in understanding between international workers (typically from the Global North) and the communities they serve (typically in the Global South) (Carpi 2021). Additionally, organisations and actors in the humanitarian sector can be exceptionally transitory and lack relevant experience [26]. Thus, ‘a major revision of humanitarian leadership and coordination of humanitarian emergencies is needed that has fewer but more competent and operational actors with a clearer command and control leadership structure’ [26], p.1). In emergency cases where other external organisations are also required it could be beneficial if the well-established organisations took responsibility for sensitive issues such as menstrual health and the smaller, newer organisations oversaw other elements that require no or minimal contact with the community.

The group being homogenous (Syrian, Muslim, Arabic-speaking) and familiar with one another aided the ease with which participants could communicate during FGDs. It also avoided issues of inclusivity or representation of differing groups. Although there is some stigma and embarrassment around menstruation in this community, it was not enough to prevent people from participating. Non-menstruators did not wish to play an active role in the project, however they supported menstruating family members to take part, posing no issue with them having private FGDs with familiar LRC staff. Non-menstruators can play an important role in either hindering or supporting a menstrual health project, both in terms of menstruator-participation within the project, or access to menstrual health after the project. Non-menstruators and/or cultural dynamics may prevent menstruators from participating in projects in the first place [14] or obstruct access to materials, services, or a supportive environment through bullying, financial control or peeping, GBV, or presence within menstruator-only facilities [10]. Thus, LRC staff knew it was important for the project to assess how non-menstruators would react so they could challenge negative attitudes and behaviours if present.

**Fig. 1:**
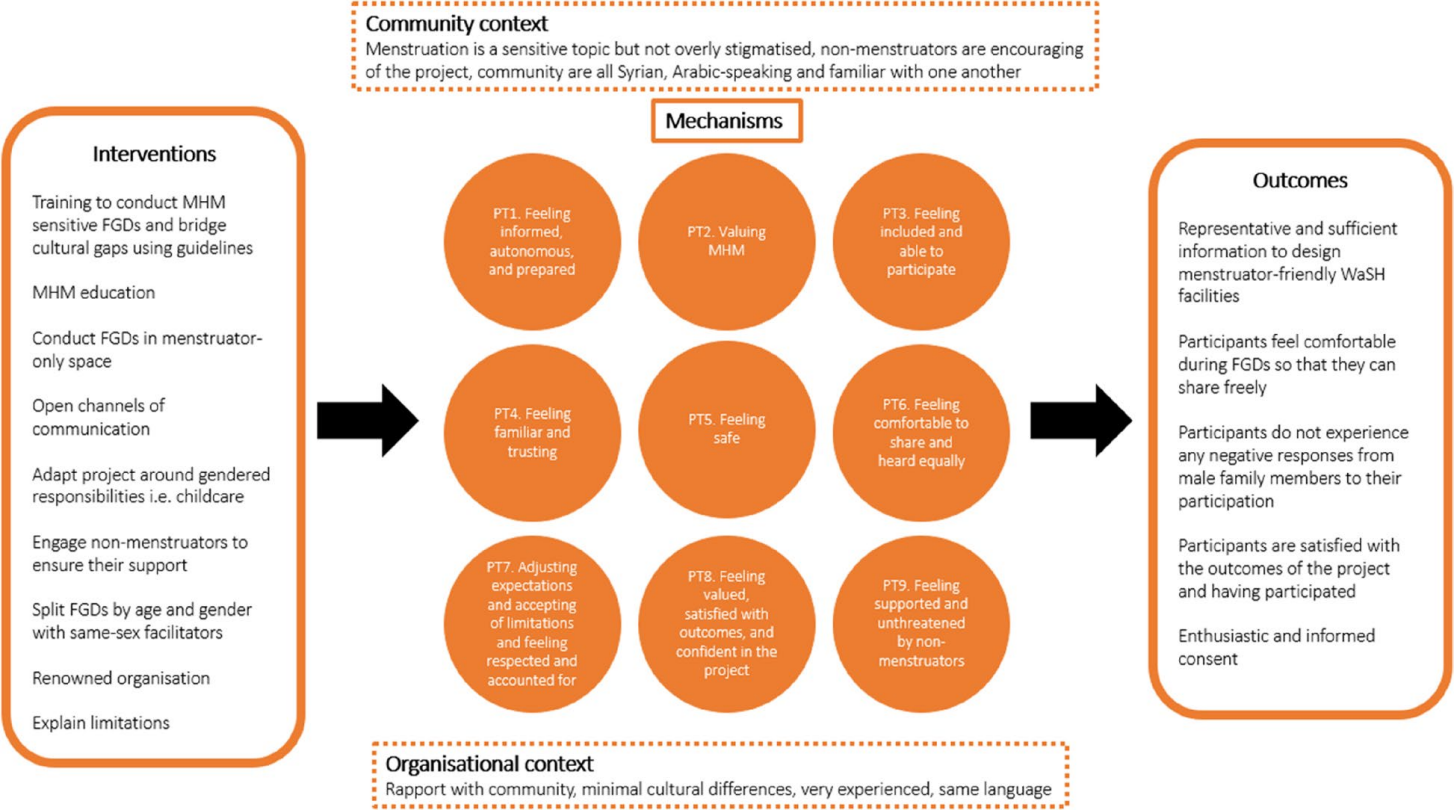
Context-Mechanism-Outcome Configurations of the refined programme theory

Figure 1 offers an explanatory framework for how these organisational and local contextual factors (top and bottom) interact with the project inputs (left) to catalyse positive feelings and reasoning that lead to the desired outcomes where participant experience is centred.

### Participatory action as a methodological tool to centre participant experience

The mechanisms explored in this paper focus on the end-user experience of participation. Here the authors discuss the difficulties of ensuring participation to be a positive and impartial practise when there exists an intrinsic power imbalance between participants and project staff. Arnstein writes that consultation can be a ‘legitimate step toward their full participation’, but when ‘not combined with other modes of participation, this rung of the ladder is still a sham since it offers no assurance that citizen concerns and ideas will be taken into account’ (1969, p.219). However, the authors argue that the level of participation required for a project is entirely dependent on its aims, and that it is sometimes not appropriate or possible for a project to achieve ‘citizen control’ (the top rung on the ladder). Instead, centring positive participant experience at all stages of the project should be one of the desired outcomes, regardless of the level of participation concerned. One universal standard is gaining the study subjects’ voluntary informed assent and consent (VIAC): ‘a process of providing potential study participants… with information about the study (e.g. risks and benefits) and allowing them to decide freely (i.e. without incentive or coercion) if they want to participate’ [29] in [3], p.814). Although Brear & Tsotetsi [3] state that ‘decolonising outcomes will be optimal’ if we implement VIAC procedures, in practice ‘power dynamics mean that consent may be neither informed nor voluntary’ (p.814). This is due to the challenge of informing participants ‘especially in postcolonial and other settings with power inequalities, because of cultural differences and social injustices, including the systematic denial of information, autonomy and rights for people with limited power’ [3], p.814). An alternative is ‘radical participatory design’ – that which ‘fully includes the community members in all activities of all phases of the design process and in all interpretation, decision-making, and planning between design activities’ [27]. This approach could work in this protracted setting as the need for humanitarianism transforms into the need for development and sustainability. However, since it takes an undefined length of time, it is not possible when a rapid response is required.

This research is useful for practitioners in the humanitarian sector who can put the described contextual and organisational factors in place before implementing the described project resources to centre participant experience alongside achieving the project’s planned outcomes. It could be beneficial to include such suggestions in an organisation’s guidance for community participation. Ultimately, this research can inform strategies for projects of a similar nature to reduce burdens on participants whilst still providing them with appropriate menstrual health facilities. Project staff felt that larger, more experienced organisations with staff who are well integrated into the community are better to conduct projects like these, coinciding with Spiegel who believes that small, inexperienced humanitarian organisations with transitory international staff is one of the ways the ‘humanitarian system is not just broke, but broken’ (2017).

Another intervention that LRC could implement is facilitating access to menstrual hygiene products – a gap raised by the menstruators. Following this project, the LRC began work with the Austrian Red Cross to install vending machines in schools that dispense menstrual products using cards, vouchers, or tokens. Another option could be to facilitate market access and cash programmes, though the efficacy of this is context dependent. In remote areas such as the one studied in this paper, it may not be viable.

### Limitations

The lead author was not able to collect the data herself in person however as FGDs were overseen by another author from the LRC this gave more autonomy to the organisation and more comfort to participants discussing the topic with familiar staff members. The Realist Evaluation was only conducted in one context as the project was only piloted in one community. With the rollout of the project into other contexts, more evaluations can be undertaken to compare how the differing contextual factors interact with the project strategies. The MCMOs that require further study before conclusions can be made are the need to engage non-menstruators in the project, the influence of having mixed nationalities and cultures within the community, and the different types and stages of an emergency.

There is always a power divide between service providers and service recipients, so the LRC WaSH officer leading data collection could have influenced participants’ willingness to freely share their perspective. As discussed in the positionality and reflexivity statement, having embedded researchers from the IFRC, BRC, and LRC may have hindered unbiased information provision and evaluation of the project.

There were only cisgender women in the community meaning that these results may not be generalizable to settings where other gender identities are present. Although the facilities were designed to be inclusive of physical disabilities, everyone in the settlement was able-bodied so this element of the facility could not be tested.

## Conclusion

This paper explored the mechanisms and contextual factors necessary for centring participant experience when implementing IFRC’s adaptable manual for designing menstruator friendly WaSH facilities in humanitarian settings. Using the IFRC and LRC’s project as an example, the research evaluated how to put participants’ needs first and ensure they face no negative implications of participating in the project, during recruitment, participation itself and after the project has finished. The programme theory developed in this study ascertained causal mechanisms that explain how organisations can work to ensure a positive participatory experience for menstruators: feeling informed, autonomous, and prepared; Valuing MHM; Feeling included and able to participate; Feeling familiar and trusting; Feeling safe; Feeling comfortable to share and heard equally; Adjusting expectations and feeling accepting of limitations as well as respected and accounted for; Feeling valued, satisfied with outcomes, and confident in the project; Feeling supported and unthreatened by non-menstruators. The results enhance understanding of the potential for wider contextual and organisational structures and resources to enrich or constrain end-user experience during participation. These were: menstruators’ valuing of menstrual health, aided by MHM education and explanations of the project details prior to recruitment, non-menstruator and religious leader support for the project, the splitting of demographic groups for FGDs, cultivating good relationships with participants, minimising cultural gaps, adapting the project around gendered responsibilities, negotiating needs into designs, explaining limitations, and creating safe spaces for FGDs. The research can inform organisational policy design in other humanitarian contexts on how to centre participant experience, wherein participants feel valued, trusting and safe at all stages of the participant process. Future research should test this explanatory framework in other humanitarian settings, which have differing contextual factors.

## Data Availability

The datasets used and/or analysed during the current study available from the corresponding author on reasonable request.
